# Retraction: HOXB13, a Target of DNMT3B, Is Methylated at an Upstream CpG Island, and Functions as a Tumor Suppressor in Primary Colorectal Tumors

**DOI:** 10.1371/journal.pone.0276670

**Published:** 2022-10-19

**Authors:** The PLOS ONE Editors

Following the publication of this article [1], the Ohio State University investigated the results presented in Fig 1 and concluded that the Fig 1A panel was produced by merging and reorientation of data from multiple different experiments, performed months apart, creating a composite Fig that did not represent the experimental conditions accurately. Following these findings, the institutional Committee recommended retraction of this article [1].

In addition to the institute’s findings, editorial reassessment of the article also raised concerns regarding the results presented in Fig 2B and Fig 3. Specifically, when adjusting the colour levels to visualise the background, there appear to be horizonal and vertical irregularities in the background of multiple panels presented in Fig 2B and Fig 3.

The authors provided repeat experiment data for the results presented in Fig 1A. However, these data were not sufficient to resolve the concerns pertaining to image fabrication that were confirmed by the institutional Committee. The original data underlying Figs 1, 2, and 3 either have not been provided for editorial review or are no longer available due to the time elapsed since the original experiments were conducted. In the absence of the original underlying data, the concerns with these Figs cannot be resolved.

In light of the inappropriate image manipulations affecting the Fig 1A results, and the unresolved concerns affecting multiple Fig 2B and Fig 3 panels that question the integrity of these data, the *PLOS ONE* Editors retract this article.

TM, CP, PY, and STJ agreed with the retraction. KG and SM responded but expressed neither agreement nor disagreement with the retraction. RC, HK, JD, SB, AM, and TH either did not respond directly or could not be reached. KG, TM, and STJ stand by the article’s findings.
